# *FLNA* mutations in surviving males presenting with connective tissue findings: two new case reports and review of the literature

**DOI:** 10.1186/s12881-018-0655-0

**Published:** 2018-08-08

**Authors:** Elyssa Cannaerts, Anju Shukla, Mensuda Hasanhodzic, Maaike Alaerts, Dorien Schepers, Lut Van Laer, Katta M. Girisha, Iva Hojsak, Bart Loeys, Aline Verstraeten

**Affiliations:** 1 Center of Medical Genetics, Faculty of Medicine and Health Sciences, University of Antwerp and Antwerp University Hospital, Prins Boudewijnlaan 43, 2650 Antwerp, Belgium; 2 Department of Medical Genetics, Kasturba Medical College, Manipal University, Manipal, India; 3 Department of Endocrinology, Metabolic Diseases and Genetics, University Clinical Center Tuzla, Children’s hospital, Tuzla, Bosnia and Herzegovina; 4 Referral Center for Pediatric Gastroenterology and Nutrition, Children’s Hospital Zagreb, University of Zagreb, School of Medicine, Zagreb, Croatia; 5 Department of Human Genetics, Radboud University Nijmegen Medical Center, Nijmegen, The Netherlands

**Keywords:** Periventricular nodular heterotopia, Live-born males, Filaminopathy, Connective tissue disease

## Abstract

**Background:**

Mutations in the X-linked gene filamin A (*FLNA*), encoding the actin-binding protein FLNA, cause a wide spectrum of connective tissue, skeletal, cardiovascular and/or gastrointestinal manifestations. Males are typically more severely affected than females with common pre- or perinatal death.

**Case presentation:**

We provide a genotype- and phenotype-oriented literature overview of *FLNA* hemizygous mutations and report on two live-born male *FLNA* mutation carriers. Firstly, we identified a de novo, missense mutation (c.238C > G, p.(Leu80Val)) in a five-year old Indian boy who presented with periventricular nodular heterotopia, increased skin laxity, joint hypermobility, mitral valve prolapse with regurgitation and marked facial features (e.g. a flat face, orbital fullness, upslanting palpebral fissures and low-set ears). Secondly, we identified two *cis*-located *FLNA* mutations (c.7921C > G, p.(Pro2641Ala); c.7923delC, p.(Tyr2642Thrfs*63)) in a Bosnian patient with Ehlers-Danlos syndrome-like features such as skin translucency and joint hypermobility. This patient also presented with brain anomalies, pectus excavatum, mitral valve prolapse, pulmonary hypertension and dilatation of the pulmonary arteries. He died from heart failure in his second year of life.

**Conclusions:**

These two new cases expand the list of live-born *FLNA* mutation-positive males with connective tissue disease from eight to ten, contributing to a better knowledge of the genetic and phenotypic spectrum of *FLNA*-related disease.

**Electronic supplementary material:**

The online version of this article (10.1186/s12881-018-0655-0) contains supplementary material, which is available to authorized users.

## Background

**Fig. 1:**
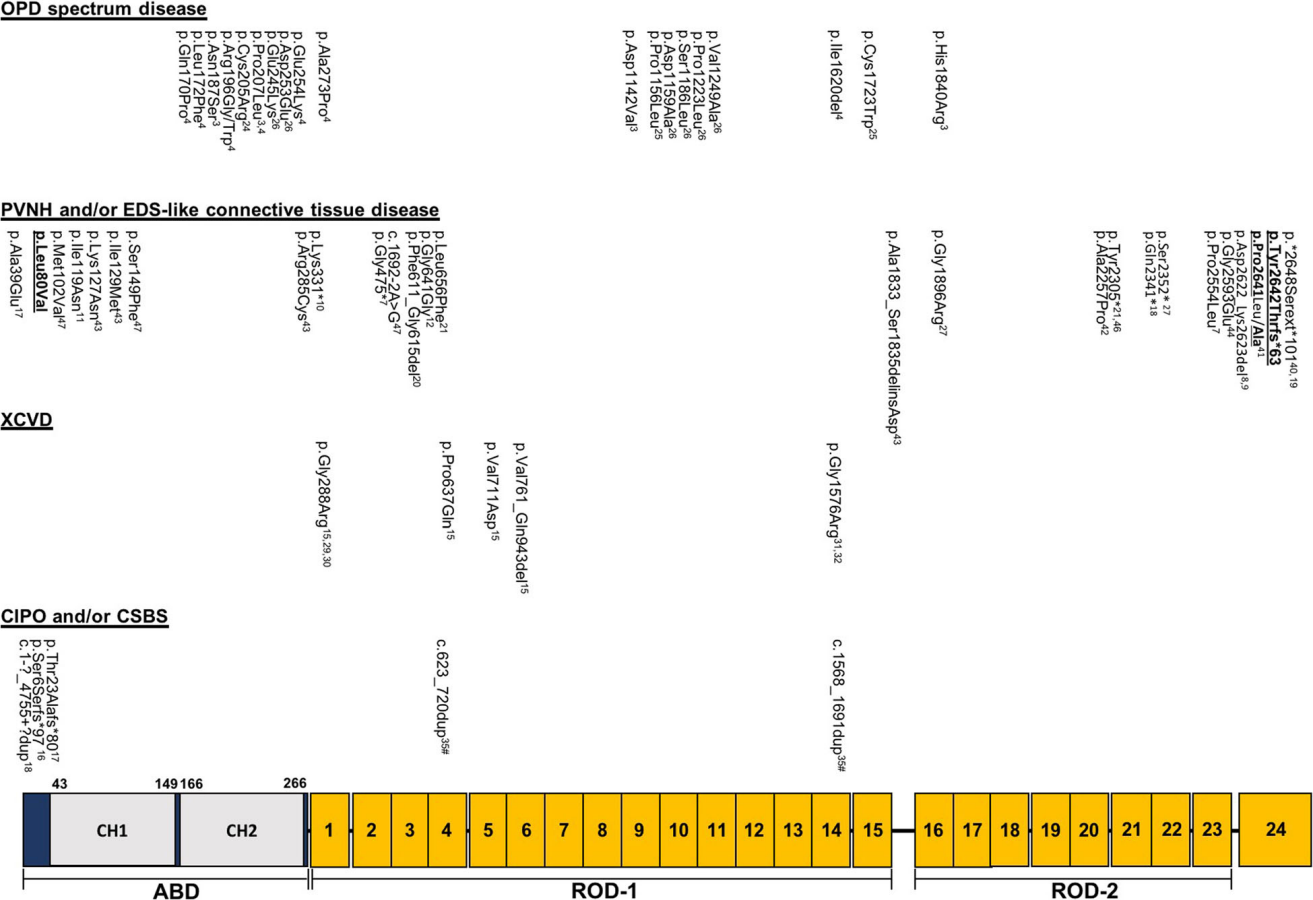
Schematic representation of the FLNA domains and repeats and overview of *FLNA* mutations and their associated disorders. Each FLNA-related disorder and their causal mutations in live-born males is depicted separately. The four diseases indicate the primary clinical expression of the patients. Mutations in bold and underlined are identified within the current paper. Mutations indicated with # occur together in patients of the same family

*FLNA* encodes a widely expressed 280-kD dimeric protein that crosslinks actin filaments into three-dimensional networks and attaches them to the cell membrane. Each monomeric chain of the protein consists of four major domains: a N-terminal F-Actin-Binding Domain (ABD) consisting of two tandem calponin homology domains (CH1 and CH2), two ROD regions which are composed of 23 Ig-like repeats, and a repeat at the C-terminus, undergoing dimerization prior to interaction with membrane receptors (Fig. 1) [1]. FLNA is involved in various cell functions, such as signal transduction, cell migration and adhesion [2] and *FLNA* mutations have been linked to a wide spectrum of disorders.

Classification of these filaminopathies depends on the nature of the underlying mutation mechanism. Gain-of-function mutations cause otopalatodigital disorders (OPD) [3, 4], while loss-of-function mutations result in periventricular nodular heterotopia (PVNH) with or without connective tissue findings [5–14], X-linked cardiac valvular dystrophy (XCVD) [15], or gastrointestinal diseases such as chronic intestinal pseudo-obstruction (CIPO) and congenital short bowel syndrome (CSBS) [16–18]. Patients presenting phenotypical features of multiple *FLNA*-related diseases have been described as well [19, 20]. While in heterozygous females the *FLNA-*related phenotype ranges from absence of overall symptoms to severe manifestations, most male mutation carriers die prenatally or in the first years of life [21–23]. This points towards a key role for FLNA in human embryonic development. In the literature, 65 different *FLNA* mutations have been reported in live-born males (Fig. 1). Here, we report on two male cases with connective tissue disease and brain abnormalities who carry novel *FLNA* mutations. In addition as an introduction, we provide a literature overview on *FLNA* genetic variability in live-born males.

### Literature overview

The OPD spectrum encompasses five X-linked disorders, in order of severity: OPD1, OPD2, frontometaphyseal dysplasia (FMD), Melnick-Needles syndrome (MNS), and terminal osseous dysplasia with/without pigmentary defects (TOD(PD)). These syndromes are predominantly characterized by skeletal dysplasia (i.e. bowing of long bones, limb deformation, and short stature), hearing loss, facial dysmorphism, cleft palate and abnormalities of the extremities. The central nervous system, cardiovascular system, gastrointestinal tract, ocular system, cutaneous system and respiratory airways are occasionally affected as well [4]. MNS or TOD(PD) male offspring of affected mothers die prenatally or shortly after birth [4]. OPD2 males die in their first year of life, mostly due to pulmonary hypertension caused by thoracic and lung hypoplasia [23, 24], while FMD exhibits a milder phenotype in affected males [25, 26]. OPD1 males survive to normal adulthood. OPD-causing mutations (missense mutations and small in-frame deletions) cluster in four specific domains, i.e. the ABD and filamin repeats 3, 10 and 14/15 (Fig. 1) [1, 4]. They act through a gain-of-function mechanism by establishing abnormal interactions between FLNA and its binding partners, such as membrane receptors, transcription factors and enzymes. No genotype-phenotype correlation has yet been described for the OPD subtypes.

PVNH is a neuronal migration disorder that can occur in *FLNA* males [21], but is more frequently observed in females. About 90% of PVNH patients present with difficult-to-treat seizures, manifesting from early childhood to adulthood [27]. It can occur with or without Ehlers-Danlos syndrome-like (EDS-like) connective tissue anomalies such as joint hypermobility, skin hyperelasticity, translucent skin and cardiovascular abnormalities. The condition is prenatally or neonatally lethal when caused by truncating *FLNA* mutations, such as N-terminal nonsense or out-of-frame splicing mutations [21]. In PVNH males who survive past infancy, distal truncating, hypomorphic missense or mosaic mutations are identified, implying that at least some functional FLNA is produced [19, 21].

XCVD is characterized by stenosis, regurgitation or prolapse of the mitral and/or aortic valve. Male XCVD cases with *FLNA* mutations typically display severe phenotypes such as polyvalvular disease, regularly requiring valve replacement surgery [28]. Although sudden cardiac death occasionally occurs during infancy, they tend to survive up to adulthood [15, 20, 28–30]. Brain imaging in a subset of cases did not reveal PVNH [28]. XCVD-causing *FLNA* mutations are mostly missense or splice-altering mutations affecting highly conserved amino acids in five of the protein’s first seven filamin repeats, i.e. repeats 1, 4, 5, 6 and 7 (Fig. 1) [15, 28–30]. Of note, two *FLNA* missense mutations (p.(Gly1554Arg) and p.(Gly1576Arg)) have most recently been linked to an X-linked syndrome of cardiac valvulopathy, but also keloid scarring, reduced joint mobility and a large optic cup-to-disc ratio [31–33]. In case of p.(Gly1554Arg), also Ebstein anomaly segregated with the mutation [33]. Further investigation is needed to determine these mutations’ precise mode of action. Besides in heart and brain, *FLNA* is highly expressed in neurons of the enteric system. As a consequence, intestinal abnormalities have recurrently been described in *FLNA* mutation-positive males (Table 1), but these usually do not present as primary symptoms. Few exceptions can be found in literature and those have been described as CIPO or CSBS (Fig. 1) [16, 17]. CIPO is characterized by severely impaired gastrointestinal motility owing to impaired involuntary or coordinated muscular contraction of the gastrointestinal tract. CSBS patients present with abdominal pain and diarrhea due to a shortened small intestine and intestinal malrotation. Initially, CIPO and CSBS mutations were identified between FLNA’s first two methionines. More recently, duplications of multiple FLNA repeats have also been described (Fig. 1) [16, 17, 34, 35].

**Table 1:** Clinical characteristics of male *FLNA* mutation-positive survivors with PVNH and/or EDS-like connective tissue disease

Mutation (AA)		Age	Age of death	PVNH	Other neurological findings	Aortic dilatation	Other cardiac involvement	Skeletal findings	Skin findings	Gastro-intestinal involvement	Other	Reference
1	*p.Ala39Glu*	37y	ND	Yes	Transient neonatal convulsions Mega cisterna magna	No	MVP with mild MR TAV with mild AR	No	No	ND	Bilateral epicanthic folds Telecanthus Anteverted nared Anteverted helices	[11]
2	*p.Leu80Val*	5y	ND	Yes	ND	No	MVP with MR TVP with TR	Bilateral hip dislocation Joint hypermobility Brachydactyly Genu recurvatum	Skin laxity Excess skin folds over the fingers	ND	Low set, posteriorly rotated ears Telecanthus Epicanthal folds Periorbital fullness Infraorbital creases	This report
3	*p.Met102Val*	3y	ND	Yes	Cerebellar hypoplasia Mildly delayed milestones	No	PDA	ND	ND	ND	Cryptorchidism	[47]
4	*p.Ile119Asn*	ND	57y	Yes	Mega cisterna magna	Yes (60 mm)	MVP with MR	ND	ND	ND	Thrombopenia	[11]
5	*p.Lys127Asn*	15y	ND	Yes	Mega cisterna magna	Yes (31 mm)	Mild MVP Thick MV	Joint hypermobility (8/9) Mild pectus carinatumScoliosis	Soft, mildly hyperelastic skin Umbilical hernia	ND	ND	[43]
6	*p.Ile129Met*	1d	ND	Yes	ND	No	Dysplastic MV and TV	ND	Skin laxity	ND	High arched palate	[43]
7	*p.Ser149Phe*	38y	ND	Yes	Complex partial and generalized seizures	No	AR	ND	ND	ND	ND	[47]
8	*p.Arg285Cys*	10y	ND	Yes	Seizures	No	MVP, small ASD	Joint hypermobility (6/9)	Thin,translucent, elastic skin Supraumbilical hernia	ND	High arched palate	[43]
9	p.Lys331* (mosaic)	1d	ND	Yes	Cerebral vasculature dysplasia Corpus callosum hypoplasia	No	PDA, VSD	ND	ND	Intestinal pseudo-obstruction (IPO) Malrotation Short gut syndrome with dilated small intestine	Bifid uvula Persistent Thrombocytopenia	[10]
10	p.Gly475*	17y	ND	Yes	ND	ND	ND	ND	ND	ND	ND	[7]
11	*c.1692-2A > G (mosaic)*	49y	ND	Yes	Cerebellar hypoplasia Partial complex and generalized seizures	Yes	AV replacement at 26y	Pes cavus	ND	ND	Bifid epiglottis	[47]
12	*p.Phe611_Gly615del*	18y	50y	ND*	Spinal osteo-arthritis Muscle hypotonia	No	Severe polyvalvular heart dysplasia Dilatation of both atria Chronic heart failure	Joint hypermobility (8/9) Scoliosis Genua valga, Bilateral varus deformity	Soft and doughy skin Skin laxity Atrophic scar Prominent veins	ND	Dysmorphic facial features	[20]
13	*p.Gly641Gly*	1d	ND	Yes	ND	No	Septal defect PVP Dysplastic TV	Talipes equinovarus	Inguinal hernia	Severe constipation Malrotation	Hypertelorism Downslanting palpebral fissures Low set, posteriorly rotated ears	[12]
14	*p.Leu656Phe*	ND	ND	Yes	Seizures	ND	ND	ND	ND	ND	ND	[21]
15	*p.Ala1833_Ser1835delinsAsp*	1d	2 m	Yes	Corpus callosum hypoplasia Cervical syringomyelia	Yes	Dysplastic aorta, MV and TV ASD, small VSD, PDA Tortuous supra-aortic vessels Pulmonary hypertension with heart failure	Joint hypermobility	Skin laxity	Malrotation (autopsy)	ND	[43]
16	*p.Gly1896Arg (mosaic)*	57y	ND	Yes	Seizures Mega cisterna magna	No	ND	ND	ND	ND	Progressive obstructive lung disease	[27]
17a	*p.Glu2142AlafsTer22*	19y	ND	No	No	Yes	Severe MR with MVP Moderate AR Dilatation of the bilateral pulmonary arteries	Pectus excavatum	Bilateral inguinal hernias Thin skin	Intestinal malrotation CIPO Crohn’s disease	Thrombocytopenia	[34]
17b		11y	ND	No	No	No	Moderate MR with MVP ASD	No	Bilateral inguinal hernias	Intestinal malrotation CIPO	Cryptorchidism	
18	*p.Ala2257Pro*	16y	ND	Yes	Moderate weakness and atropy of the intrinsic hand muscles and wrist extensors bilaterally Seizures	No	PDA AV dysfunction	Joint hypermobility (7/9) Pectus excavatum Bilateral genu recurvatum	Skin laxity	Malrotation Short gut syndrome	ND	[42]
19a	p.Tyr2305*	ND	ND	Yes	Seizures	ND	ND	ND	ND	ND	ND	[21, 46]
19b		36y	36y	Yes	Seizures	Yes	AR Left ventricular hypertrophy Left atrial enlargement	ND	ND	ND	ND	
20	p.Gln2341*	Prenatally	6w	Yes	Posterior fossa arachnoid cyst	ND	ND	Spina bifida occulta Proximally placed thumbs	ND	Diaphragmatic defect Displaced stomach and spleen Short gut syndrome with dilated loops of small intestine	“Square face”, flat philtrum Wide metopic suture High-arched palate	[18]
21	p.Ser2352* (mosaic)	38y	ND	Yes	Inward rotation of anterior ventricular horns Corpus callosum hypoplasia Mega cisterna magna	ND	ND	Broad and flattened OPD I-like end phalanges of both feet	ND	ND	Retrognathia Hypertelorism Low-set ears	[27]
22	*p.Pro2554Leu*	56y	ND	Yes	ND	ND	ND	ND	ND	ND	ND	[7]
23	*p.Gly2593Glu*	ND	ND	Yes	Seizures Migraines	No	ND	Joint hypermobility Tall thin habitus	ND	ND	Left-sided sensorineural hearing loss Retinal lattice degeneration High arched palate	[44]
24a	*p.Asp2622_Lys2623del*	5y	ND	Yes	Corpus callosum hypoplasia Mega cisterna magna	ND	ND	Scoliosis Mild platyspondyly Spatulate finger tips Short, broad phalanges and metacarpus Skeletal dysplasia of the posterior fossa and frontal sinuses Delayed bone age	ND	ND	Dysmorphic facial features	[8, 9]
24b		Neonatal	ND	Yes	Corpus callosum hypoplasia Mega cisterna Magna	ND	ND	Mild platyspondyly, Spatulate finger tips Short broad phalanges and metacarpus Skeletal dysplasia of the posterior fossa and frontal sinuses Delayed bone age	ND	Malrotation	Dysmorphic facial features	
25	*p.Pro2641Ala* *p.Tyr2642ThrfsTer63*	1d	2y	ND*	Broad interhemispheric fissures and subarchnoid spaces with echogenic parenchyma Muscle hypoplasia and hypotonia	No	Dysmorphic/elongated cusps TV with TVR Thinned/elongated cusps MV with MVP, ASD Dilated pulmonary arteries Pulmonary hypertension, leading to heart failure and early demise	Joint hypermobility Pectus excavatum	Translucent skin	Chronic diarrhea	Dysmorphic facial features Food allergies Recurrent bronchitis	This report
26a	*p.Pro2641Leu*	1d	ND	Yes	Enlarged anterior fontanel Ventriculomegaly Seizures Mental retardation	No	PDA	ND	ND	ND	Severe bronchodysplasia	[41]
26b		1d	8 m	Yes	Enlarged anterior fontanel Ventriculomegaly Microgyria Microcephaly	No	PDA	ND	ND	ND	Severe bronchodysplasia resulting in death	
27a	p.*2648Serext*101	Neonatal	ND	Yes	Corpus callosum hypoplasia Retrocerebellar cyst	No	PDA	ND	Inguinal hernia	Pyloric stenosis Constipation Malrotation Short gut syndrome	Dysmorphic facial features	[40, 19]
27b		Neonatal	ND	Yes	Mildly delayed motor development – hyptonia Retrocerebellar cyst	No	Mildly dysplastic MV PDA	Mild pectus excavatum	ND	Severe constipation Gastro-intestinal obstruction Short gut syndrome	Recurrent upper respiratory tract infections Dysmorphic facial features	
27c		1d	ND	Yes	Hypoplastic cerebellum and vermis	No	ASD	ND	ND	Short gut syndrome Malrotation	Dysmorphic facial features	

*AR* aortic regurgitation, *ASD* atrial septal defect, *AV* aortic valve, *MVR*: mitral valve regurgitation, *MR* mitral regurgitation, *MV* mitral valve, *MVP* mitral valve prolapse, *ND* not described, *PDA* patent ductus arteriosus, *PVP* pulmonary valve prolapse, *TAV* tricuspid aortic valve, *TV* tricuspid valve, *TVP* tricuspid valve prolapse, *TVR* tricuspid valve regurgitation, *VSD* ventricular septal defect, *CIPO* chronic intestinal pseudo-obstruction. Patient 23a is brother of patient 23b

## Case presentations

### Proband with connective tissue findings carrying a de novo p.(Leu80Val) mutation

#### Clinical description

**Fig. 2:**
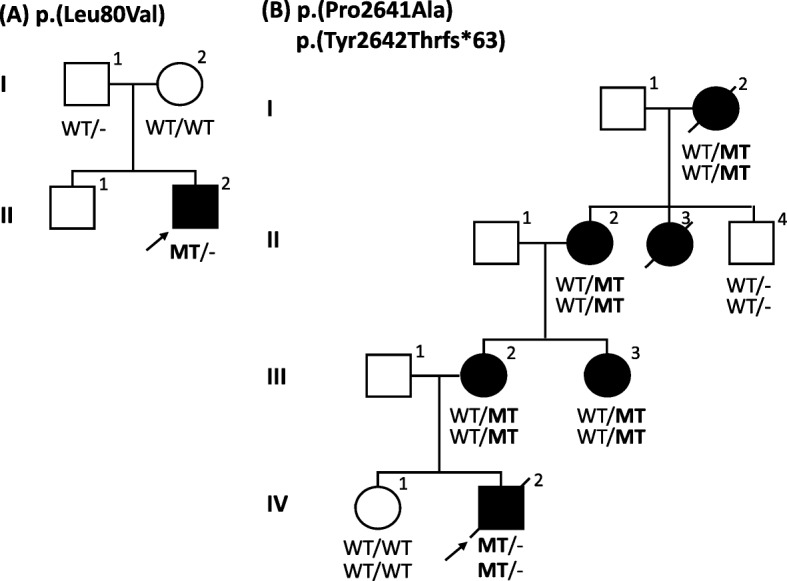
Family pedigrees with their respective mutations. Squares represent males, circles represent females, black-filled symbols represent affected individuals and a + or − sign denotes the presence or absence of *FLNA* mutations. **a** Pedigree of case A, the Indian patient (proband) with a de novo *FLNA* missense mutation p.(Leu80Val) and his unaffected family members. **b** Pedigree of case B, the Bosnian patient with a frameshift p.(Tyr2642Thrfs*63) and a missense mutation p.(Pro2641Ala) in *FLNA*

**Fig. 3:**
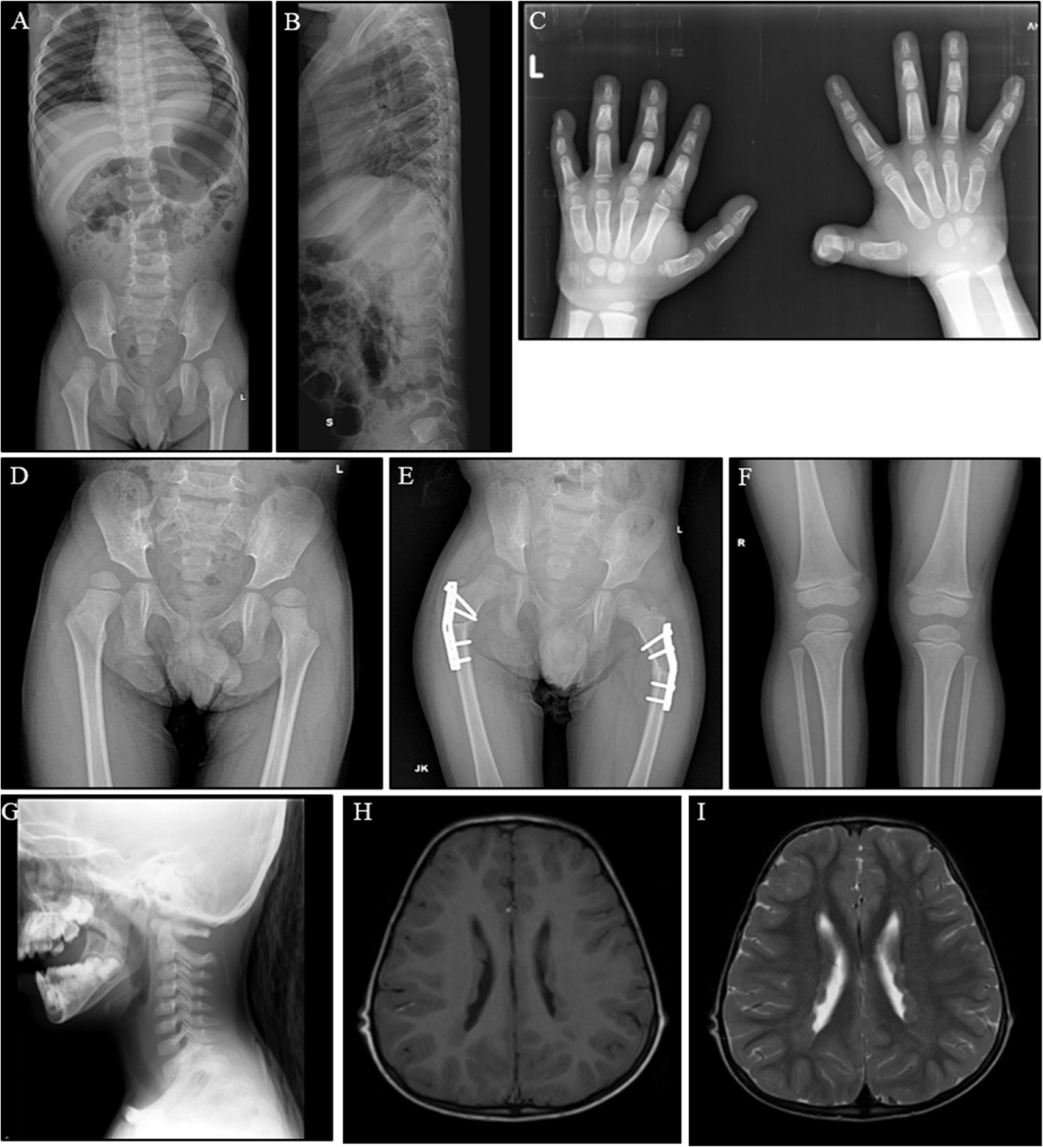
Skeletal survey of five-year old Indian patient (case A). **a**, **b**, **g** At 5 years of age the skeletal survey revealed tall vertebral bodies, **c** delayed ossification of carpal bones, dislocated terminal phalanx of right thumb, **d**, **e** bilateral hip subluxation for which he underwent bilateral varus derotation osteotomy, **f** genu recurvatum and (**h**, **i**) magnetic resonance imaging of the brain showed PVNH along the subependymal regions of both lateral ventricles

**Fig. 4:**
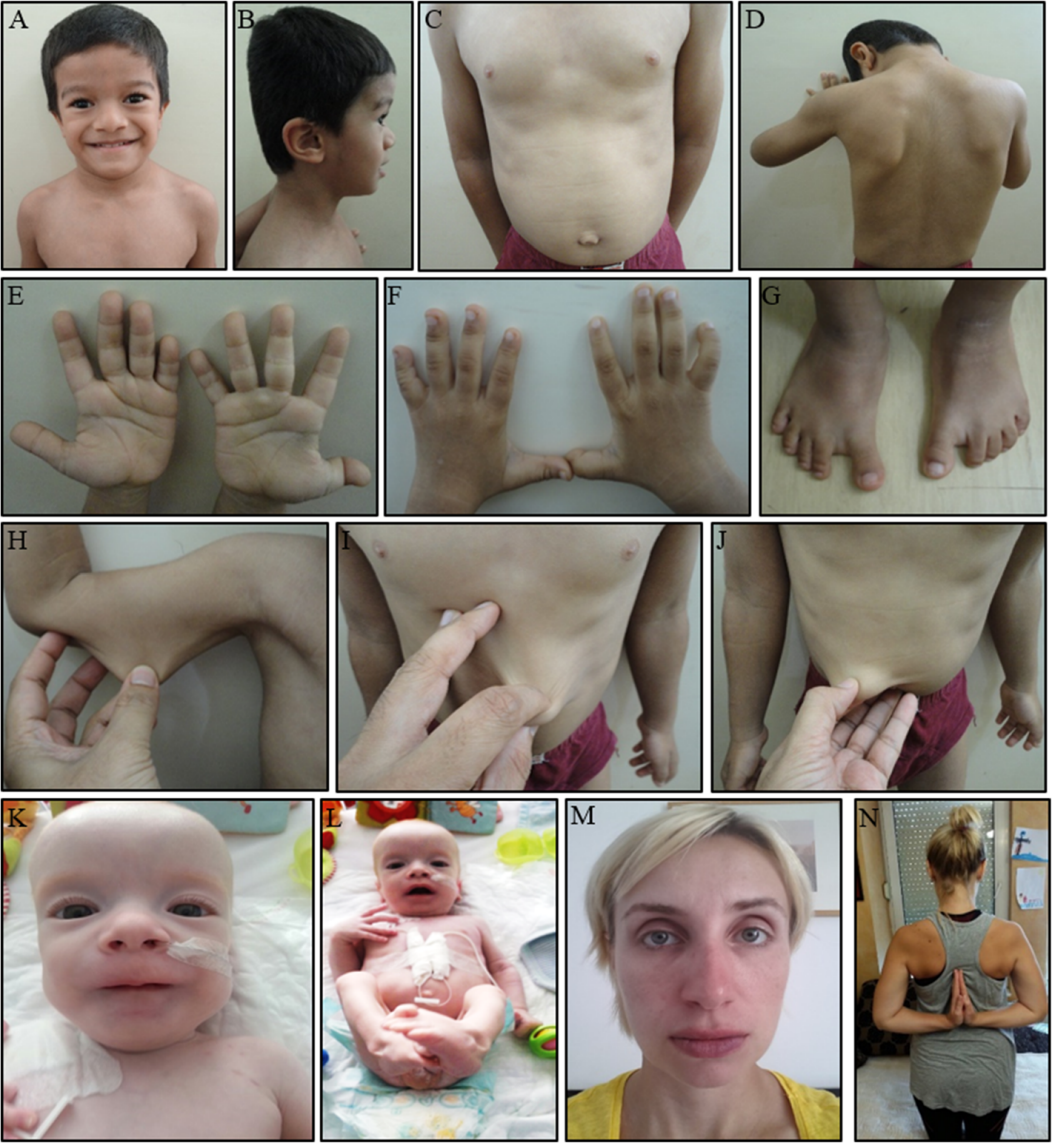
Clinical features. In the five-year old Indian patient (case A) the following features were noted: (**a**) flat face, telecanthus, orbital fullness, upslant of palpebral fissures, prominent nasolabial folds, (**b**) brachycephaly, midface retrusion and low-set ears, (**e**, **f**) mild brachydactyly and a deformed right thumb due to an unstable and lax interphalangeal joint, (**g**) broad and medially deviated great toes with sandal gap and flat feet and (**c**, **d**, **h**, **i**, **j**) extra skin folds and significant skin laxity. The proband of family B presented with (**k**, **l**) hypertelorism, pectus excavatum, clubfeet and hypermobile joints whereas the mother (III-2) demonstrated hypertelorism and joint hypermobility (**m**, **n**)

The proband is a five-year old Indian boy (II-2) without a family history of connective tissue or cardiovascular disease (Fig. 2a). He was born at 34 weeks of gestation to non-consanguineous parents by normal vaginal delivery with a birth weight of 2.4 kg. Bilateral hip dislocation and cryptorchidism were noted on the second day of life. A pavlik harness was applied during the first six months and he underwent bilateral varus derotation osteotomy for hip subluxation (Fig. 3e) at four years of age. Cryptorchidism was surgically treated at three years of age. At the age of five, his height was 98 cm (2 SD below the mean), head circumference was 49.5 cm (2 SD below the mean) and weight was 14 kg (2 SD below the mean). Craniofacial examination demonstrated brachycephaly, telecanthus with upslanting palpebral fissures, epicanthal folds, periorbital fullness and infraorbital creases bilaterally and low-set, posteriorly rotated ears (Fig. 4a, b). Further clinical examination revealed marked skin laxity with excess skinfolds over the fingers (Fig. 4c, d, h, i and j). Increased mobility across all joints, and brachydactyly with proximally placed thumbs (Figures e,f), flat feet with sandal gap and medially deviated great toes were also observed (Fig. 4g). Radiographs revealed dislocated distal phalanx of the right thumb (Fig. 3c). The pelvis had narrow iliac bones and a wide femoral neck (Fig. 3d). The spine showed tall vertebral bodies (Fig. 3a, b, g). Motor development was mildly delayed, illustrated by the fact that he only started walking independently at 20 months of age. At age 5, he presented with genu recurvatum and bilateral hip subluxation for which he underwent bilateral varus derotation osteotomy (Fig. 3d, e, f). Cognitive and language milestones as well as his ophthalmological parameters were normal. Echocardiography revealed a myxomatous and prolapsed mitral valve with moderate regurgitation. Additionally, it also showed tricuspid aortic valve prolapse with mild regurgitation. Aortic measurements were within the normal range. After molecular diagnosis, magnetic resonance imaging (MRI) of the brain showed PVNH along the subependymal regions of both lateral ventricles (Fig. 3h, i; Additional file 1: Table S1).

#### Molecular diagnosis

After obtaining informed consent of the parents, genomic DNA of the proband (III-2) was screened for mutations in 37 connective tissue disease genes (Division of Medical Genetics, Charité, Berlin), including the known genes for cutis laxa. No pathogenic variants were identified. Whole exome sequencing of III-2 was performed, which led to the identification of a novel missense mutation (c.238C > G, p.(Leu80Val)) in *FLNA*. Genotyping of p.(Leu80Val) in the proband’s parents revealed that the mutation arose de novo*.* In silico analysis strongly supports variant pathogenicity: (1) the variant is absent in the gnomAD database (http://gnomad.broadinstitute.org/; including 1000 Genomes database and the ExAC database) [36, 37] and also absent from the dbSNP database (build 147) [38] (2) p.(Leu80) is highly evolutionary conserved (down to zebrafish) and locates to the protein’s F-actin-binding domain, and (3) prediction programs SIFT, PolyPhen2 and MutationTaster predict a damaging effect on protein structure and/or function.

### An EDS-like patient with two mutations in *cis*: a frameshift p.(Tyr2642Thrfs*63) and a missense mutation p.(Pro2641Ala)

#### Clinical description

The second patient is a male Bosnian infant with a positive family history for connective tissue and cardiovascular disease (Fig. 2b). He was born at 37 weeks of gestation by Caesarean section for placenta previa after an uneventful pregnancy. Immediately after birth he received oxygen support because of an underdeveloped left lung. Neonatal jaundice was treated with phototherapy. In the second week of life he was hospitalized again because of bacterial sepsis. The proband displayed dysmorphic features, such as hypertelorism, pectus excavatum, clubfeet and features of connective tissue disease, such as translucent skin, bilateral inguinal hernia and hypermobile joints (Fig. 4k, l). His neurological examination was significant for muscle hypotonia and poor muscle mass. Other health issues included hydro-ureteronephrosis, hypospadias, food allergies, chronic diarrhea (which improved with a modified diet) and recurrent bronchitis. Echocardiographic examination showed elongated cusps of the tricuspid valve with regurgitation, thinned and elongated cusps of the mitral valve with mitral valve prolapse, dilation of the pulmonary arteries with pulmonary hypertension and atrial septum defect with right atrial and ventricular dilatation. Aortic measurements at 12 months of age were normal. Brain ultrasound showed broader interhemispheric fissures and subarachnoid spaces with echogenic parenchyma. Since only ultrasound evaluation of the brain was available of the patient, no solid conclusion can be made about the presence or absence of PVNH. He died due to severe pulmonary hypertension and heart failure in his second year of life.

Family history revealed significant clinical findings in several female members (Fig. 2b). The mother (III-2) (Fig. 4m, n) presented with joint hypermobility and mild skin hyperelasticity. Her cerebral computed tomography (CT) and MRI-scan revealed cerebral atrophy, subependymal nodular heterotopia in the wall of the lateral ventricles and right frontal horn and intracranial hyperostosis of the frontal bones. Echocardiography of the mother (III-2) showed insufficiency of the aortic and pulmonary valves at age 33 years (both grade I-II). Hypermobile joints in the maternal aunt (III-3) and grandmother (II-2) were also noted. His great-aunt (II-3) had joint hypermobility, thin and soft skin and mild aortic valve regurgitation (diagnosed at age 9, but stable). She developed epilepsy with partial seizures from age 22. A brain MRI showed partial agenesis of the corpus callosum, cerebellar hypoplasia and PVNH. She died at the age of 48 due to an accident, which was a consequence of a seizure. The great-grandmother (I-2) was described as a frail person who died at age 82 years with renal insufficiency and heart failure (Additional file 2: Table S2).

#### Molecular diagnosis

Based on the patient’s phenotype, a Marfan syndrome (MFS) diagnosis was initially suspected. Mutation analysis, including multiplex ligation-dependent probe amplification (MLPA) of *FBN1* revealed no pathogenic variants. Further genetic investigation involved mutation analysis of the coding sequences of 22 genes [39] known to be implicated in the etiology of cardiovascular disease using gene panel sequencing. Two hemizygous *cis* variants in exon 48 of *FLNA* were identified and, subsequently, validated with Sanger sequencing: a frameshift c.7923delC; p.(Tyr2642Thrfs*63) and a missense c.7921C > G; p.(Pro2641Ala) mutation. For both mutations, several lines of evidence suggest involvement in disease development. Pathogenicity of p.(Tyr2642Thrfs*63) is supported by: (1) its absence from the gnomAD database (http://gnomad.broadinstitute.org/; including 1000 Genomes database and the ExAC database) [36, 37] and also absent from the dbSNP database (build 147) [38], (2) the fact that the mutation leads to the formation of an alternative stopcodon approximately 57 amino acids downstream of the original one, resulting in an aberrant protein C-terminus (i.e. a ‘no-stop mutation’), and (3) proven pathogenicity of a previously reported frameshift mutation with a similar effect at the protein level (p.(*2648Serext*101)), leading to systemic anomalies, including CIPO, PVNH, pyloric stenosis, patent ductus arteriosus (PDA) and atrial septum defect (ASD) [19, 40]. Evidence for pathogenicity of p.(Pro2641Ala) includes: (1) its absence from the gnomAD database (http://gnomad.broadinstitute.org/; including 1000 Genomes database and the ExAC database) [36, 37] and absent from the dbSNP database (build 147) [38], (2) p.(Pro2641) is highly conserved (up to Tetraodon) and locates to the C-terminal part of the protein, which is important for FLNA dimerization, (3) causality of another amino acid substitution at the same position (c.7922C > T; p.(Pro2641Leu)), causing PVNH, PDA, severe bronchodysplasia and partial seizures [41] and (4) classification of the variant as disease causing by prediction programs PolyPhen2, SIFT and MutationTaster.

After obtaining informed consent of the family members, segregation analysis of p.(Tyr2642Thrfs*63) and p.(Pro2641Ala) was performed, revealing the presence of both mutations in the patient’s clinically affected mother (III-2), maternal aunt (III-3), maternal grandmother (II-2) and maternal great-grandmother (I-2), further demonstrating pathogenicity of the mutations. His unaffected sister (IV-1) and brother of his grandmother (II-4) were mutation-negative.

## Discussion and conclusions

About 30 male *FLNA* mutation-positive patients with PVNH have been reported, of which only six (patients 5, 6, 8, 12, 15, 18; Table 1) display both PVNH and EDS-like features (Table 1) [42–46]. Four of the latter were reported to present with cardiovascular anomalies, including aortic dilatation, mitral valve prolapse (MVP) and a dysplastic aortic, mitral or tricuspid valve [43, 45]. Joint hypermobility (5/6), skin translucency and/or hyperelasticity (5/6) were also common, while scoliosis and partial seizures were only noted once (Table 1) [42–44]. Null mutations typically lead to embryonic or early lethal PVNH in males. So far, the rare live-born males with PVNH or PVNH with EDS-like features were diagnosed with hypomorphic missense and distal truncating mutations, providing a sufficient amount of functional FLNA protein [43, 45]. Here, we report on a five-year old Indian boy who presents with connective tissue findings, attributed to a de novo *FLNA* mutation (p.(Leu80Val)) that locates to the CH1 domain. The initial presentation included skin laxity, joint hypermobility and prolapse of the mitral and tricuspid valve with moderate regurgitation. Additionally, motor development was mildly delayed and the skeletal system was prominently affected. After molecular diagnosis, brain imaging confirmed the presence of PVNH.

Untill this report, five live-born unrelated male cases had been described who were molecularly confirmed to carry missense mutations in the CH1 domain (amino acid 43–149) [43, 47, 48] (Fig. 1). Two unrelated males with *FLNA* mutations in CH1 have been described by Guerrini and colleagues [47]. The first male (p.(Met102Val)) was diagnosed with PVNH and cerebellar hypoplasia without seizures (patient 3, Table 1), while the other (p.(Ser149Phe)) had infrequent complex partial and secondarily generalized seizures (patient 7, Table 1). In both patients cardiovascular abnormalities were observed, including PDA and aortic valve insufficiency [47]. However, in contrast to our patient no connective tissue anomalies were observed. A p.(Glu82Val) mutation was previously described in a family with three affected females in two generations and five presumably affected boys whom all died within the first days or months of life. However, molecular confirmation could not be obtained for the male patients due to lack of DNA [48]. Reinstein et al. identified two unrelated PVNH males with EDS-like connective tissue manifestations who carried CH1-located *FLNA* mutations. A p.(Lys127Asn) mutation was found in a 15-year old male who had, besides PVNH without seizures, cardiovascular abnormalities (MVP with thickened mitral valve leaflets and dilatation of the sinuses of Valsalva), soft and hyperextensible skin, joint hypermobility, umbilical hernia, mild lumbar scoliosis and a spontaneous pneumothorax (patient 5, Table 1). A second male with a nearby-located *FLNA* mutation, p.(Ile129Met), presented with highly similar features, including PVNH without seizures, increased mobility across the joints, skin laxity and right inguinal hernia (patient 6, Table 1). From a cardiovascular point of view, however, he was more severely affected. He had dysplastic mitral and tricuspid valves with regurgitation, requiring mitral and tricuspid valvuloplasty at 10 months of age. He also developed recurrent pulmonary infections and at the age of one he was still hypotonic and had to be fed via a nasogastric tube (Table 1) [43]. The mutations identified by Reinstein and colleagues are located within a conserved hydrophobic region between amino acids 121–147 (Fig. 1). It is predicted that mutations within this motif have a direct effect on the actin binding of FLNA. In contrast, our mutation (affecting AA 80) is located upstream of this motif and is predicted to have an indirect effect on the actin binding capacity of the ABD [49]. This might explain the difference observed in facial and skeletal phenotype between our patient and those described by Reinstein et al. (2013).

The second case we reported is a male Bosnian patient, who carried a no-stop mutation (p.(Tyr2642Thrfs*63)) and a missense mutation (p.(Pro2641Ala)). He presented with skin and joint connective tissue findings. PVNH status is unknown as no MRI was performed. Cardiovascular anomalies included dilatation of both pulmonary arteries with pulmonary hypertension and heart failure of which he died at two years of age.

Two reports on affected males (patient 27 a,b,c; Table 1) describe a no-stop frameshift mutation (p.(*2648Serext*101)) that is highly similar to ours (Fig. 1) [19, 40]. This mutation affects the last amino acid of FLNA and is predicted to create a novel stop codon in the 3’ UTR polyadenylation signal. Besides PVNH, described male cases had prominent facial features (a low nasal bridge, broad mouth and a prominent forehead) and/or gastrointestinal problems (obstruction, intestinal malrotation and/or a short small bowel). Cardiovascular anomalies were also observed, including PDA, ASD and dysplastic mitral valves. These characteristics correspond with what is seen in our case B patient. Mildly delayed motor development was noted once. Remarkably, none presented with features reminiscent of EDS [19 and personal communication], which clearly differs from our case (case B) (Table 1).

Pulmonary artery dilation has not been commonly described in male *FLNA* mutation carriers. Only two other cases have been reported. Reinstein and colleagues examined a male *FLNA* mutation carrier (p.(Ala1833_Ser1835delinsAsp)), who presented with PVNH and pulmonary hypertension with heart failure (patient 15, Table 1). He had marked joint laxity and increased skin elasticity [43]. Another report mentioned two brothers with a 4-bp deletion (c.6425_6428delAGAG; p.(Glu2142Alafs*22)) in exon 40 of *FLNA*, predicted to cause a premature protein truncation (patients 17a and 17b, Table 1; Fig. 1). RT-PCR experiments on cDNA of the siblings suggested that the *FLNA* mutation induced both normal and alternative splicing. Both brothers had cardiac complications, with the oldest brother presenting with dilatation of the ascending aorta and bilateral pulmonary arteries. Bilateral inguinal hernias were noted in both brothers, while pectus excavatum only presented in the oldest one [34]. Those reports together with the findings in our case B indicate that pulmonary hypertension and/or dilatation are part of the phenotypic spectrum of *FLNA*-related disorders in males (Table 1).

In summary, *FLNA* can either exert loss-of-function or gain-of-function mechanisms, leading to clinically distinct disorders. Disease is typically more severe in male mutation carriers when compared to their female counterparts. Whereas most mutation-positive males die prenatally, a literature search demonstrates that a subset of them, i.e. those with hypomorphic missense, distal truncating or mosaic *FLNA* mutations, are live-born. Of these, eight (patients 5, 6, 8, 12, 15, 17a, 17b,18; Table 1) have been reported to present with EDS-like connective tissue disease. The two new cases (patients 2 and 25) reported here contribute to the genotypic and phenotypic spectrum of EDS-like connective tissue disease, expanding the list of live-born males with *FLNA* mutations.

## Additional files

Additional file 1: **Table S1.** Clinical Timeline Case A. (DOCX 16 kb)

Additional file 2: **Table S1.** Clinical Timeline Case B. (DOCX 14 kb)

## Abbreviations

ABD: F-Actin-Binding Domain
ASD: Atrial septum defect
CH: Calponin homology
CIPO: Chronic intestinal pseudo-obstruction
CSBS: Congenital short bowel syndrome
CT: Computed tomography
EDS-like: Ehlers-Danlos syndrome-like
FLNA: Filamin A
FMD: Frontometaphyseal dysplasia
MFS: Marfan syndrome
MLPA: Multiplex ligation-dependent probe amplification
MNS: Melnick-Needles syndrome
MRI: Magnetic resonance imaging
MVP: Mitral valve prolapse
OPD: Otopalatodigital disorders
PDA: Patent ductus arteriosus patent ductus arteriosus
PVNH: Periventricular nodular heterotopia
TOD(PD): Terminal osseous dysplasia with/without pigmentary defects
XCVD: X-linked cardiac valvular dystrophy
