# Supplementary material for: FLNA mutations in surviving males presenting with connective tissue findings: two new case reports and review of the literature
Source: BMC Med Genet. 2018 Aug 8;19:140. doi: 10.1186/s12881-018-0655-0 (PMC6083619; doi:10.1186/s12881-018-0655-0)
Supplement: Supplementary file 1 — Table S1. Clinical Timeline Case A. (DOCX 16 kb) [file 12881_2018_655_MOESM1_ESM.docx]

**Table S1.** Clinical Timeline Case A

| Dates | Relevant Past Medical History and Interventions | | |
| --- | --- | --- | --- |
|  | Bilateral hip dislocation, bilateral cryptorchidism, brachycephaly, telecanthus, epicanthal folds, periorbital fullness, infraorbital creases bilaterally, low set ears, skin laxity, hypermobility of joints, genu recurvatum, excess skin folds, sandal gap, flat feet, dislocated distal phalange of right thumb, tall vertebral bodies in spine, nodular heterotopia, myxomatous and prolapsed mitral valve with regurgitation, tricuspid valve prolapse with regurgitation | | |
| Dates | Summaries from initial and follow-up visits | Diagnostic Testing (including dates) | Interventions |
| First 6 months | Bilateral hip dislocation |  | Palvik harness |
| 3 years of age | Cryptorchidism |  | operation |
| 4 years of age | Bilateral varus derotation osteotomy |  |  |
