# Supplementary material for: FLNA mutations in surviving males presenting with connective tissue findings: two new case reports and review of the literature
Source: BMC Med Genet. 2018 Aug 8;19:140. doi: 10.1186/s12881-018-0655-0 (PMC6083619; doi:10.1186/s12881-018-0655-0)
Supplement: Supplementary file 2 — Table S1. Clinical Timeline Case B. (DOCX 14 kb) [file 12881_2018_655_MOESM2_ESM.docx]

**Table S2.** Clinical Timeline Case B

| Dates | Relevant Past Medical History and Interventions | | |
| --- | --- | --- | --- |
|  | Neonatal jaundice, hypertelorism, pectus excavatum, clubfeet, translucent skin, bilateral inguinal hernia and hypermobile joints, muscle hypotonia and poor muscle mass, hydro-ureteronephrosis, hypospadias, food allergies, chronic diarrhea, recurrent bronchitis, elongated cusps of the tricuspid valve with regurgitation, thinned and elongated cusps of the mitral valve with mitral valve prolapse, dilation of the pulmonary arteries with pulmonary hypertension and atrial septum defect with right atrial and ventricular dilatation, broader interhemispheric fissures and subarachnoid spaces with echogenic parenchyma, died due to severe pulmonary hypertension and heart failure in his second year of life | | |
| Dates | Summaries from initial and follow-up visits | Diagnostic Testing (including dates) | Interventions |
|  | Neonatal jaundice |  | Phototherapy |
| 7 days old | Deterioration of general condition and systemic bacterial infection |  |  |
| 2 months old | Extension pyelo-ureteric system of both kidneys | MCUG, Ultrasound, MRI |  |
| 5 months old | Cardiac anomalies | Echocardiography, CT | Aldactone 2x2mg, Enap 1x0.5 mg, Sildenafil tablets (0.25mg/kg) |
|  | Poly-valvular heart disease |  |  |
| 6 months old | broader interhemispheric fissures and subarachnoid spaces with echogenic parenchyma | Ultrasound |  |
| 7 months old | Severe growth failure | Endocrinology |  |
